# Characterisation of a common hotspot variant in acute intermittent porphyria sheds light on the mechanism of hydroxymethylbilane synthase function

**DOI:** 10.1002/2211-5463.13490

**Published:** 2022-09-26

**Authors:** Marthe S. Christie, Mikko Laitaoja, Aasne K. Aarsand, Juha P. Kallio, Helene J. Bustad

**Affiliations:** ^1^ Department of Biomedicine University of Bergen Norway; ^2^ Department of Chemistry University of Eastern Finland Joensuu Finland; ^3^ Norwegian Porphyria Centre (NAPOS), Department for Medical Biochemistry and Pharmacology Haukeland University Hospital Bergen Norway; ^4^ Norwegian Organization for Quality Improvement of Laboratory Examinations Haraldsplass Deaconess Hospital Bergen Norway

**Keywords:** acute intermittent porphyria, catalysis, haem, hydroxymethylbilane synthase, mass spectrometry, polypyrroles

## Abstract

Hydroxymethylbilane synthase (HMBS) is the third enzyme involved in haem biosynthesis, in which it catalyses the formation of tetrapyrrole 1‐hydroxymethylbilane (HMB). In this process, HMBS binds four consecutive substrate molecules, creating the enzyme‐intermediate complexes ES, ES_2_, ES_3_ and ES_4_. Pathogenic variants in the *HMBS* gene are associated with the dominantly inherited disorder acute intermittent porphyria. In this study, we have characterised the p.R26H variant to shed light on the role of Arg26 in the elongation mechanism of HMBS and to provide insights into its effect on the enzyme. With selected biophysical methods, we have been able to show that p.R26H forms a single enzyme‐intermediate complex in the ES_2_‐state. We were also able to demonstrate that the p.R26H variant results in an inactive enzyme, which is unable to produce the HMB product.

## Graphical abstract

Hydroxymethylbilane synthase normally binds four consecutive porphobilinogen substrates, creating the enzyme‐intermediate complexes ES, ES_2_, ES_3,_ and ES_4_. The pathogenic variant p.R26H forms a single enzyme‐intermediate complex in the ES_2_‐state and is blocked for turnover. High‐resolution mass spectrometry of enzyme‐intermediate complexes of wild‐type (green) and p.R26H variant (blue).

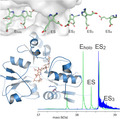

**Abbreviations**
AIP: acute intermittent porphyria
ALA: δ‐aminolaevulinic acid
CD: circular dichroism
DPM: dipyrromethane
DSF: differential scanning fluorimetry
FT‐ICR MS: Fourier transform ion cyclotron resonance mass spectrometry
HMB: 1‐hydroxymethylbilane
HMBS: hydroxymethylbilane synthase
MD: molecular dynamics
PBG: porphobilinogen
SEC: size exclusion chromatography
TEV: tobacco etched virus

Hydroxymethylbilane synthase (HMBS or porphobilinogen deaminase; PBGD) is the third enzyme in the haem biosynthetic pathway, where it catalyses the formation of the linear tetrapyrrole 1‐hydroxymethylbilane (HMB) [1, 2, 3]. In this process, four consecutive PBG molecules are bound, creating the enzyme‐intermediate complexes ES, ES_2_, ES_3_, and ES_4_ respectively, as reviewed in Phillips [4]. Briefly, the elongation extends from a dipyrromethane (DPM) cofactor, which is covalently bound to a cysteine in the active site [5, 6]. DPM is formed by two PBG molecules and acts as a primer in the elongation process [7, 8]. HMBS without bound cofactor (apo‐HMBS) has, however, higher affinity for the HMB product than the substrate PBG, indicating that ES_2_ may be formed directly from apo‐HMBS [9].

In humans, there are two tissue‐specific isoforms of HMBS derived from alternative splicing: the housekeeping (HMBS1) and the erythroid‐specific (HMBS2) enzyme, with 361 and 344 amino acids, respectively. The 17 first amino acids at the N‐terminus of HMBS1 have no known function, however, it has been suggested that they have a regulatory function or a role in e.g., protein trafficking [10, 11].

Pathogenic variants in the *HMBS* gene are associated with the low penetrant autosomal dominant disorder acute intermittent porphyria (AIP). Both HMBS isoforms are affected if the variant is in the shared part of *HMBS* because of alternative splicing [12]. In *HMBS* gene variant carriers, wild‐type (wt) enzyme expresses from one allele and the loss in total HMBS enzymatic activity can be up to 50%, depending on the variant [13]. However, many genetically predisposed carriers will never develop symptomatic disease, which is characterised by potentially life‐threatening neurovisceral acute attacks, as the associated loss of HMBS activity is not sufficient for clinical expression, and the prevalence of likely pathogenic *HMBS* variants observed in the general population greatly outnumbers symptomatic disease prevalence [14]. In symptomatic AIP patients, clinical disease is associated with situations that increase the demand for haem, leading to an upregulation of δ‐aminolaevulinic acid (ALA) synthase 1, the first enzyme in the haem biosynthesis, whereupon the affected HMBS enzyme becomes rate‐limiting [4]. In this setting, porphyrin precursors upstream from HMBS – ALA and porphobilinogen (PBG) – are produced in excess in the liver [15]. The overproduction and consequently accumulation of neurotoxic precursors are considered to be the cause of acute attacks. Increased levels of ALA and PBG in urine are used to diagnose AIP and other acute porphyrias in which ALA and PBG accumulate. AIP is associated with a varying natural history, from never‐symptomatic carriers to patients with recurrent severe acute attacks. There is also a high phenotypic heterogeneity, even within the same family. Clinically, acute attacks are characterised by automatic dysfunction including severe abdominal pain, tachycardia, hypertension, and a wide range of neurological and psychiatric symptoms [15, 16, 17]. Acute attacks are triggered by various factors, most importantly drug‐induced hepatic haem depletion via induction or suicidal inactivation of cytochrome P450 enzymes (CYPs) [18], hormonal changes associated with the menstrual cycle [19], low‐calorie intake [20, 21], infection and stress [22, 23], though often a clear triggering factor cannot be established. Additionally, *HMBS* variant carriers are at risk of long‐term complications including primary liver cancer, hypertension, and kidney failure [24, 25, 26, 27]. Sporadic acute attacks are treated by supportive measures and haem infusions, where the latter downregulates the hepatic ALAS1 activity through negative feedback. Currently, the only cure for AIP is liver transplantation [28], however, a recently introduced RNA interference therapy, Givosiran, has been shown to be effective in reducing attacks in patients with recurrent acute attacks [29, 30].

More than 500 different *HMBS* variants associated with clinical disease, have been published [31]. However, for many variants, the effect on the HMBS enzyme has not been clearly described and the knowledge of the HMBS mechanism at the molecular level is still limited. Even with the numerous HMBS crystal structures and molecular dynamic (MD) simulations published in the last decades, the exact mechanisms of polypyrrole elongation are not yet completed through to the stage of Michaelis complex EP [32]. From the earliest studies, it was recognised that the enzyme in the crystal had to be with the cofactor in its active form [33].

Arg26 has been suggested to have a crucial role in the elongation process. Its location in hydrogen‐bonding distance to the proposed substrate entry site (yellow, Fig. 1A), close to the available substrate and “growing end” of the intermediate complex where it can interact with the acetate of PBG, indicates a role in both positioning and possibly deaminating the incoming substrates [34, 35]. The substitution of arginine 26 to histidine (Fig. 1B) results in a disease‐associated variant (c.77G>A; p.R26H), which has been described in several different AIP families [36, 37]. Two other missense substitutions at the same location, p.R26C and p.R26L, have also been reported [38, 39, 40, 41, 42, 43, 44, 45]. In this work, we have sought to characterise the p.R26H variant to shed light on Arg26 and its role in the elongation mechanism of HMBS. Using a combination of anion‐exchange chromatography, native PAGE, circular dichroism (CD), differential scanning fluorimetry (DSF), and high‐resolution mass spectrometry, we demonstrate how the variant is trapped in the ES_2_‐state, and that it is inactive without product turnover. Characterising HMBS variants in depth can provide an insight into their effect on the enzyme's structure and function, thereby providing more detailed information on the HMBS mechanism, allowing for a better understanding of genotype–phenotype correlations, and potentially providing useful information for future enzyme‐specific treatment options.

**Fig. 1:**
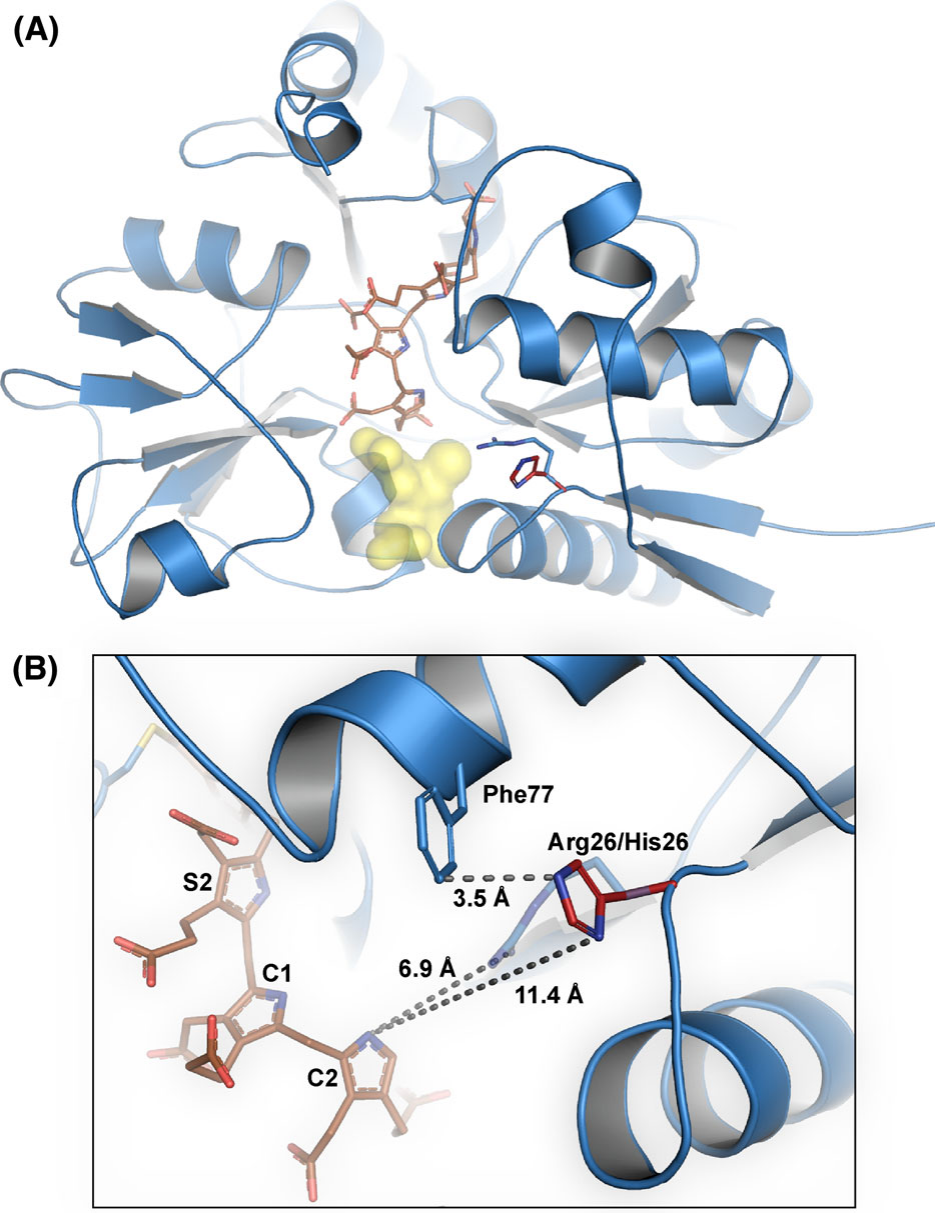
Cartoon illustration of the hypothetical structure of p.R26H‐HMBS in the ES_2_ state. The figure is based on the structure of the p.R173W variant (PDB ID: 7AAK), prepared in the pymol molecular graphics system, Version 2.5.2, Schrödinger, (LLC, New York, NY, USA). His26 has been modelled simply by substituting the Arg26 side chain using the most common side chain rotamer of histidine. The ES_2_ substrate unit is in brown and His26 in dark red. The putative substrate entry site is shown in yellow, also representing the “growing end” on the C2 ring of the intermediate complex. (A) The overall structure of HMBS enzyme in the ES_2_ state. (B) Close‐up for Arg/His26 showing the distances to bound PBG and putative packing of His26 against Phe77.

## Materials and methods

Human wt‐HMBS and variant were expressed in *Escherichia coli* BL21(DE3) strains as His‐tagged fusion proteins with a tobacco etched virus (TEV) protease cleavage site (NLYFQ/G) in a pET‐28a(+)‐TEV vector. In this study, the erythroid‐specific (HMBS2) enzyme was used but the numbering corresponding to the housekeeping isoform (HMBS1) was kept to match the reports from previous studies: p.R9H (c.26G>A) in the presented sequence equals to p.R26H (c.77G>A). HMBS expression constructs were purchased from GenScript Biotech (Piscataway, NJ, USA), with the following sequence for the variant:

(G)MRVIRVGT**H**KSQLARIQTDSVVATLKASYPGLQFEIIAMSTTGDKILDTALSKIGEKSLF
TKELEHALEKNEVDLVVHSLKDLPTVLPPGFTIGAICKRENPHDAVVFHPKFVGKTLETLPEK
SVVGTSSLRRAAQLQRKFPHLEFRSIRGNLNTRLRKLDEQQEFSAIILATAGLQRMGWHNRVG
QILHPEECMYAVGQGALGVEVRAKDQDILDLVGVLHDPETLLRCIAERAFLRHLEGGCSVPVA
VHTAMKDGQLYLTGGVWSLDGSDSIQETMQATIHVPAQHEDGPEDDPQLVGITARNIPRGPQL
AAQNLGISLANLLLSKGAKNILDVARQLNDAH

Theoretical masses: HMBS_apo_: 37 735.94 Da; ES_2_ 38573.22 Da; ES_3_: 38 782.29 Da.

### Protein expression and purification

Wt‐HMBS and variant p.R26H were expressed in *E. coli* BL21 (DE3). Fifty millilitre Lennox LB EZmix™ (Merck KGaA, Darmstadt, Germany) starter cultures supplemented with 50 μg·mL^−1^ kanamycin were grown overnight in a shaking incubator at 200 r.p.m. and 30 °C. The starter cultures were diluted in 1 L LB broth supplemented with kanamycin and incubated further at 200 r.p.m. and 37 °C until OD_600_ of 0.5–0.6 before induction with 0.5 mm IPTG and further incubated overnight at 200 r.p.m. and 27 °C. Cells were harvested at 6000 ***g*** for 30 min and pellets were resuspended in 15 mL wash buffer: 400 mm NaCl, 25 mm Tris at pH 8, 20 mm imidazole at pH 7.5, 0.5 mm tris (2‐carboxyethyl) phosphine (TCEP) added protease inhibitors (1 tablet cOmplete™ w/EDTA (Roche Diagnostics GmbH, Mannheim, Germany), 10 mm benzamidine and 0.2 mm PMSF). Resuspended pellets were frozen at −20 °C until use.

The resuspended pellet was thawed on ice and lysis buffer was added to 40 mL before sonication on ice for 3 × 2 min at 25 W output with 10 s pulses and pauses on ice, then centrifuged at 20 000 ***g*** for 45 min. The supernatant was loaded onto a nickel‐charged affinity resin (Ni‐NTA Agarose; Qiagen, Hilden, Germany) equilibrated in wash buffer, using a gravity flow purification column for His‐tag affinity chromatography. The resin was washed 3 × CV with wash buffer before eluting the protein with 400 mm NaCl, 25 mm Tris at pH 8, 400 mm imidazole at pH 7.5 and 0.5 mm TCEP.

Eluted His‐tagged HMBS was added 1 : 100 TEV protease to cleave off the fusion tag, during dialysis with 400 mm NaCl, 25 mm Tris pH 8 and 0.5 mm TCEP in a cellulose membrane dialysis tubing with MW cut‐off at 14 000 ON at 4 °C. Cleaved protein was run through Ni‐NTA pre‐equilibrated with 150 mm NaCl, 25 mm Tris pH 8 and 0.5 mm TCEP, collected and concentrated using Amicon Ultra centrifugal 30 kDa cut‐off filters (Merck) at 4000 ***g***. NanoDrop spectrophotometer (Thermo Fisher Scientific Inc., Waltham, MA, USA) and sequence‐derived extinction coefficients were used to determine protein concentrations.

Concentrated wt‐HMBS and p.R26H were further purified by size exclusion chromatography (SEC) on a GE HiLoad Superdex 75 16/60 PG column connected to an Äkta Pure Protein Purification System (Cytiva Europe GmbH, Freiburg, Germany) at 4 °C, using 30 mm NaCl, 25 mm Tris pH 8 (buffer A) at flow rate 1 mL·min^−1^.

### Anion‐exchange chromatography

Anion‐exchange chromatography using a Mono Q 4.6/100 column (Cytiva) connected to an Äkta Pure (Cytiva) was used to separate the enzyme‐intermediate complexes. The Mono Q column was equilibrated in buffer A at 4 °C. The protein was eluted in a gradient from 0% to 65% using 400 mm NaCl, 25 mm Tris pH 8 (buffer B) at 4 °C and peak fractions were collected. The isolated intermediates were concentrated using Amicon Ultra centrifugal 30 kDa cut‐off filters (Merck) for further analyses.

### Polyacrylamide gel electrophoresis

Proteins were analysed by native polyacrylamide gel electrophoresis (native‐PAGE) with a loading buffer of 125 mm Tris–HCl, 40% glycerol, 0.002% bromophenol blue. The samples were loaded on a 10% Mini‐PROTEAN^®^ TGX™ Precast Protein Gel (Bio‐Rad Laboratories, Hercules, CA, USA) and ran for 2½ h at 140 V and 4 °C. The gel was stained with Coomassie blue staining and visualised by Molecular Imager ChemiDoc XRS+ with imagelab software (Bio‐Rad).

### Circular dichroism spectroscopy

Far‐UV spectra were obtained using a J‐810 Jasco spectropolarimeter (Jasco Europe S.R.L., Cremella, Italia) with a CDF‐426S Peltier element (Jasco) for temperature control and a 300 μL quartz cuvette with a path length of 1 mm. Wt‐HMBS and p.R26H at 5 μm were prepared in 10 mm K_2_HPO_4_, 100 mm NaF. Three scans were obtained for each spectrum and the buffer scans were subtracted using the integrated Spectra Manager^TM^ software. DichroWeb and the CDSSTR analysis method with reference set 3, were used for secondary structure predictions [46, 47].

### Enzymatic activity

The enzymatic activity was assayed by adding 2 μg enzyme to 50 mm Tris–HCl at pH 8 and pre‐incubating the sample for 3 min at 37 °C. To start the enzyme reaction 100 μm prewarmed PBG was added, and the reaction was stopped by adding 5 m HCl and 0.1% benzoquinone after 4 min. Benzoquinone oxidised the produced uroporphyrinogen I into uroporphyrin in the dark on ice for 30 min. The sample was subsequently centrifuged to remove precipitated protein, before the absorbance of uroporphyrin was measured at 405 nm (ε = 548 000 m ^−1^·cm^−1^) using a NanoDrop spectrophotometer. HMBS activity was expressed as nmol uroporphyrinogen I per hour per mg under the given conditions. Michaelis–Menten kinetics was used to determining the kinetic parameters; *K*_m_ and *V*_max_ were obtained using a range of PBG concentrations from 3.125–2000 μm and analysed using the Michaelis–Menten enzyme kinetics model in graphpad prism (GraphPad Prism version 9.2.0 for MacOS; GraphPad Software, San Diego, CA, USA, www.graphpad.com).

### Mass spectrometry

Prior to mass spectrometric experiments, the enzyme samples were buffer‐exchanged to 20 mm NH_4_OAc pH 6.8 using PD Miditrap G‐25 column (Cytiva). Their concentrations were determined by UV absorbance at *A*_280_ using their sequence‐derived extinction coefficient (15 470 m ^−1^·cm^−1^). Further dilutions were made using HPLC quality acetonitrile, water, and acetic acid. Mass spectra were measured using a Bruker 12‐T solariX XR FT‐ICR mass spectrometer (Bruker Daltonik GmbH, Bremen, Germany). The substrate reactions were undertaken using Bruker timsTOF mass spectrometer (Bruker Daltonik). All samples were measured by direct infusion, at 2 μL·min^−1^, using standard electrospray source. The instrument was calibrated using NaPFHA clusters before measurements. The mass spectrometer was operated using ftmscontrol 2.2 software, and the data were processed using the bruker dataanalysis 5.1 software. Deconvolution (i.e., zero‐charge) spectra were calculated using a Maximum Entropy (maxent) deconvolution algorithm, built into the software. All masses are reported as most abundant isotopic masses.

### Differential scanning fluorimetry

A Light Cycler 480 Real‐Time PCR (Roche) was used to obtain thermal denaturing scans. Five micromolar protein was prepared in phosphate‐buffered saline with 5× SYPRO Orange (Agilent Technologies, Santa Clara, CA, USA) and analysed in 384‐well plates, as previously described [48]. Unfolding curves were recorded at a scan rate of 2 °C·min^−1^ with 0.2 °C intervals from 20 °C to 99 °C. Data were analysed using htsdsf explorer [49].

## Results

In this work, we have utilised the recombinant HMBS wt and variant p.R9H, both in the human HMBS2 isoform (NM_001024382.2:c.26G>A), which corresponds to p.R26H in the HMBS1 isoform (NM_000190.4:c.77G>A). There are minimal structural and no known functional differences between these two isoforms [11]. To keep consistency with the literature, we will refer to the constructs in this work as wt‐HMBS and the variant as p.R26H.

**Fig. 2:**
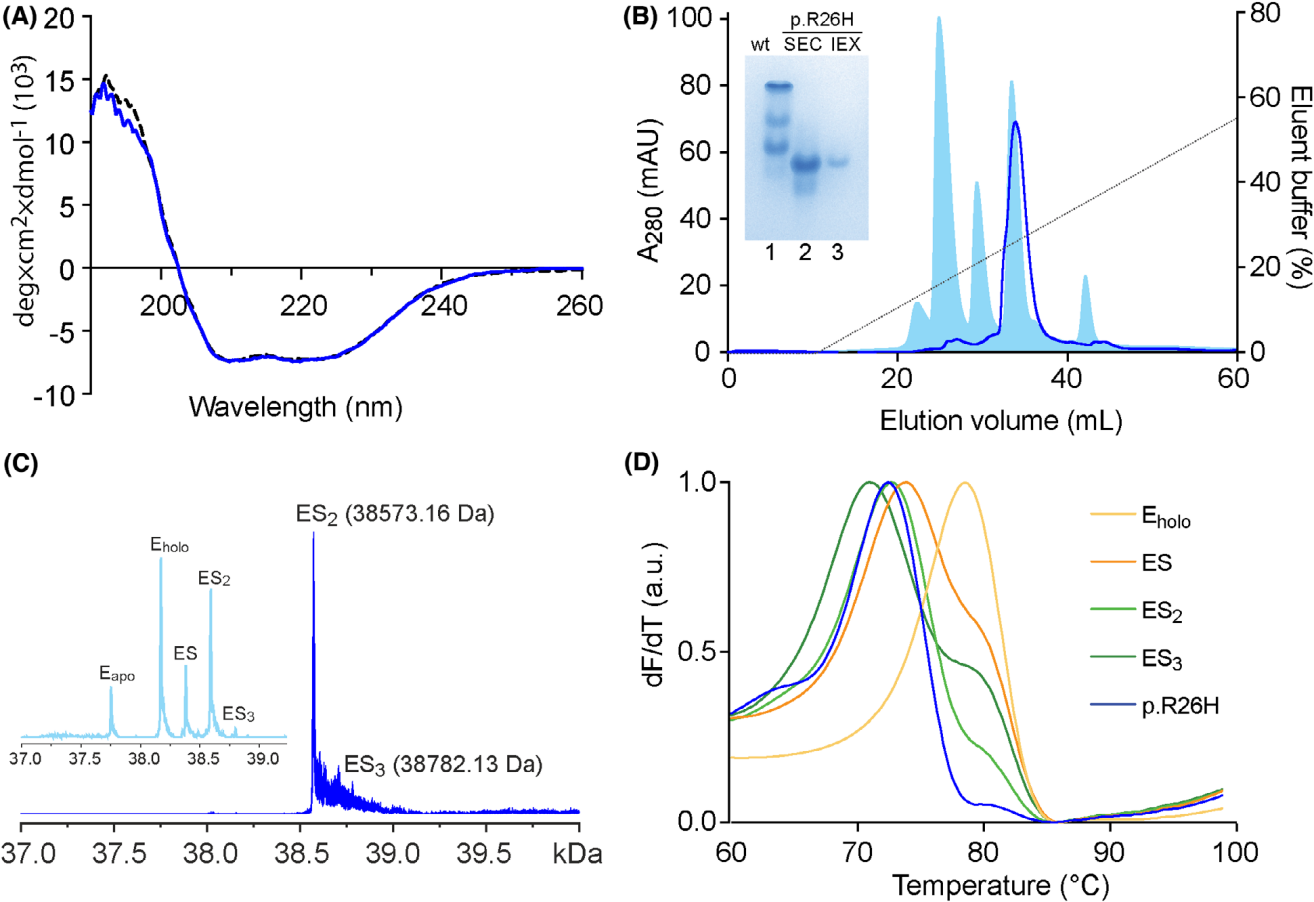
Biophysical characterisation of HMBS variant p.R26H. Purification and detection of enzyme‐intermediate complexes of wt‐ and p.R26H HMBS. (A) Far‐UV CD spectroscopy of HMBS. 0.2 mg·mL^−1^ wt‐HMBS (grey solid line) and p.R26H variant (blue line) analysed in triplicates, corrected for concentration. (B) Isolation of enzyme‐intermediate complexes. Recombinantly expressed wt‐HMBS (light blue) and p.R26H (blue) enzyme‐intermediate complexes are separated and isolated using anion‐exchange chromatography. Inset: Native‐PAGE analysis of wt‐HMBS (lane 1), p.R26H from SEC (lane 2), and the isolated main peak of p.R26H intermediate from anion‐exchange chromatography (IEX, lane 3). (C) Charge‐deconvoluted ESI FT‐ICR spectrum of p.R26H. The p.R26H variant was measured in denaturing conditions at 1 μm protein concentration. The variant was detected mainly as ES_2_ (38 573.16 Da) and there was small amounts of ES_3_ (38 782.13 Da. Inset: Purified wt‐HMBS enzyme showing a mixture of intermediates (E_apo_, E_holo_, ES, ES_2_ and ES_3_). (D) Thermal denaturation of p.R26H monitored by DSF. The first derivative of unfolding as a function of temperature is shown as the average of four in‐plate replicates. The intermediates from wt‐HMBS are E_holo_ (yellow), ES (orange), ES_2_ (light green), and ES_3_ (dark green). The p.R26H variant is shown in blue.

The recombinant p.R26H was successfully purified, in amounts similar to the wt‐HMBS. CD spectroscopy was performed prior to further experiments to confirm secondary structural composition and fold. A comparison of wt‐HMBS and p.R26H (Fig. 2A) clearly indicated the correct folding of the variant, which was further clarified by the estimation of the secondary structural content (Table S1).

Purified HMBS can be further separated into its enzyme intermediates' complexes, using anion‐exchange chromatography with a buffer gradient [11, 48, 50]. Comparing results for wt‐HMBS and p.R26H, it was evident that p.R26H mainly eluted as a single intermediate at ~ 26% of gradient buffer B (Fig. 2B), corresponding to ES_2_ in wt‐HMBS. Native PAGE has commonly been used to demonstrate the enzyme‐intermediate complexes, because of the difference in net charge between them [11, 48, 51]. As seen in Fig. 2B, lane 1, all the expected bands from wt‐HMBS were observed. On the other hand, p.R26H migrated as one major band (Fig. 2B, lane 2), with minor bands corresponding to the smaller peaks in the chromatogram (Fig. 2B). The isolated main peak of p.R26H is shown in Fig. 2B, lane 3. The migration pattern of p.R26H was inconclusive, as the major peak migrated further than the ES_2_ of wt‐HMBS. Using Fourier transform ion cyclotron resonance mass spectrometry (FT‐ICR MS), the intermediate complex distribution in both wt‐ and variant HMBS was analysed, as previously reported [52]. FT‐ICR MS measurements in denaturing conditions verified the findings for p.R26H, being mainly present in the intermediate ES_2_‐state (Fig. 2C). The tailing of the ES_2_ peak was most likely caused by incomplete desalting. Uroporphyrinogen was also observed (Fig. S1A), indicating that this cyclic product has affinity for and can be co‐purified with the enzyme [9]. Uroporphyrinogen I or III can be derived from either recombinant HMBS or endogenous *E. coli* HMBS enzyme activity, respectively. The native‐like conditions confirmed that the Arg‐to‐His substitution did not cause any unfolding of the enzyme (Fig. S1B). As expected, intermediates corresponding with E_holo_, ES, ES_2_ and ES_3_ were detected in wt‐HMBS (Fig. 2C, inset and Fig. S2A).

Wt‐HMBS demonstrated a specific activity of 2249 ± 38 nmol·mg^−1^·h^−1^, however, p.R26H was completely inactive and incapable of producing the HMB product. Hence, the kinetic parameters of the variant could not be determined, whereas we obtained a *V*_max_ of 2996 ± 9 nmol·mg^−1^·h^−1^ and *K*_m_ of 28 ± 1 μm for wt‐HMBS (Fig. S3). FT‐ICR MS showed a rapid formation (~ 1 min) of the ES_4_ intermediate when wt‐HMBS was incubated with 10× PBG (Fig. S2B), and after 60 min the intermediate distribution reappeared, indicating depletion of the substrate (Fig. S2C). The addition of 10× PBG to p.R26H and incubation up to 24 h did not change the distribution (Fig. S2D), further corroborating that the p.R26H is inactive without the capacity of turnover.

We have previously demonstrated that the thermostability of HMBS decreases with the number of bound PBG substrates [48]. We wanted to investigate whether the Arg‐to‐His substitution had an impact on the structural stability, or if the effect could be attributed solely to the change in amino acid. Therefore, we analysed the thermostability of the isolated wt and variant enzyme‐intermediate complexes, using DSF. The wt‐HMBS intermediates demonstrated a loss in the half‐denaturing temperature (*T*_m_) represented as maxima of the first derivative (d*F*/d*T*) from 78.6 ± 0.1 °C to 71.0 ± 0.2 °C for E_holo_ and ES_3_, respectively (Fig. 2D). The *T*_m_‐value of the isolated intermediate in p.R26H variant was 72.6 ± 0.1 °C, corresponding to the ES_2_ of wt‐HMBS, which was 72.8 ± 0.1 °C.

## Discussion

Arg26 is one of many conserved arginine residues across species and has been implicated to be critical for HMBS catalysis [53, 54]. In crystal structures PDB IDs 1PDA and 3ECR, Arg26 has been observed in hydrogen‐bonding distance to an acetate or sulphate ion (derived from solutions used in the crystallisation process) located in the predicted substrate‐binding site [34, 55]. Other crystal structures such as PDB IDs 5M6R and 7AAK also indicate several intradomain interactions for Arg26, such as Pi‐cation interaction with Phe77, hydrophobic alkyl interaction with Ala31 and Leu81, hydrogen‐bond to Thr25 and electrostatic interactions with Asp99 [11, 52], indicating its important location in the active site, and putative role in both enzyme activity and structural stability. Sato et al. [50] demonstrated that Arg26, Arg167 and Arg173 all interact with the substrate analogue positioned in the substrate‐binding site. Interestingly, previously described variants p.R167W and p.R173W, both interacting with the propionate side chain of PBG, exhibit different effects, where the first can form all enzyme‐intermediate complexes, whereas the latter creates a catalytic blockage and a subsequent accumulation of a single intermediate [52]. Our results demonstrate that p.R26H also results in a variant that is catalytically blocked with ES_2_ as the accumulated intermediate, however, no major structural changes are indicated based on secondary structure content and thermostability analysis.

Arg26 in humans corresponds to Arg11 in *E. coli* HMBS. The p.R11H and p.R11L variants expressed in *E. coli* were reported as correctly folded but unable to bind substrate [54, 56]. Interestingly, two previous reports on the human p.R26H variant have incongruent observations, with one suggesting altered interaction with the cofactor and the other no pyrrole chain elongation [43, 57]. However, we do not have direct evidence of the formation of ES_2_ from ES, and cannot rule out that endogenous HMBS produces HMB product that interacts directly with apo‐variant resulting in the ES_2_ complex without involving Arg26 proton donation, as has also been suggested for variants of D84 in *E. coli* [9].

Recent advances in the field include the crystal structure of wt‐HMBS in its ES_2_‐state (PDB ID: 5M6R), demonstrating how the cofactor‐binding loop with the Cys261 residue that anchors the cofactor, is pulled backwards with the two incoming PBG substrates [11]. The two new substrate molecules replace the cofactor in their positions and interactions [11, 52]. Furthermore, our report on the p.R173W‐variant trapped in the ES_2_‐state (PDB ID: 7AAK) revealed that Arg173 is an essential residue for the enzyme to move from ES_2_ to ES_3_ in the elongation process [52]. Despite providing new structural information, neither of these reports, naturally, could offer any light on details beyond the ES_2_‐state, or the role of Arg26 in the elongation process. Nevertheless, the critical function of Arg26 in enzymatic catalysis has been described based on docking and MD simulations, which suggested Arg26 as the most likely proton donor in the forming of ES and ES_2_, but too far away for the next step from ES_2_ to ES_3_ and beyond [53]. However, it was assumed that the elongation process extended from the cofactor without involving a rearrangement of the cofactor‐binding loop [53], a movement that has later been demonstrated in other reports [11, 52]. Our current results support that Arg26 is essential for the formation of the intermediates up to the ES_2_ state, possibly in concert with Arg173, where Arg173 likely acts as the coordinator for the bound PBG also stabilising the structure, and where Arg26 has the role as proton donor for upcoming substrates. Both p.R173W and p.R26H are trapped in the ES_2_‐state, however, neither have, in our studies, responded to the addition of excess substrate. Where p.R173W displays some structural instability as a function of temperature [48], p.R26H resembles wt‐HMBS in the ES_2_‐state in its reaction to temperature. This could be explained by the direct interaction between Arg173 and the bound PBG, where the Arg → Trp substitution disturbs the stabilising hydrogen‐bond network, which connects the structural domains via the bound PBG, and yields a lowered thermostability, whereas the interactions that are lost when Arg26 is replaced by His has little or no impact on the structural stability. In addition, the similar secondary structure content between wt‐HMBS and p.R26H indicates that the overall secondary structure is not affected by the introduction of the histidine. Modelling of the variant based on existing high‐resolution ES_2_‐structure (PDB ID: 7AAK) was therefore seen as sufficient to illustrate the p.R26H structure (Fig. 1).

Bung et al. [53] also predict, via MD simulations, that Arg26 interacts with both Thr58 and Asp61. Asp61 is located in the active‐site loop and is a highly conserved residue, predicted to have important interactions contributing to the stability of the structure [58]. Interestingly, Arg173 is also in the vicinity of Asp61 considering the flexible nature of the active‐site loop, and it could be speculated that these three residues act in concert. Based on structural evidence, the interactions between Arg26 and the active‐site loop (residues Ser57‐Lys74) can only occur when the is loop completely closed as shown in the HMBS structure from *Arabidopsis thaliana* (PDB ID: 4HTG) [59]. In this structure, the cofactor is in its oxidised form. Thus, we cannot conclude that the closing of the active‐site loop occurs as a part of the elongation process. However, p.D61N, p.D61H, and p.D61Y are known pathogenic variants of Asp61 [60, 61, 62] indicating the importance of the loop and these residues for the function of the enzyme.

The p.R26H variant has been reported in several different families with AIP, originating in different countries and spanning several continents. In AIP, there is a large molecular heterogeneity and most variants are restricted to single or just a few families, but the p.R26H occurs at a known hotspot involving CpG dinucleotides [63] and has also been described to occur *de novo* [36, 37]. Most publications on p.R26H have diagnostic data, but there is relatively little clinical data included and thus any assessment of severity such as early symptom onset or clinical penetrance is therefore not possible. However, generally, few genotype–phenotype correlations have yet been established in AIP and only null‐allele variants have been described to be associated with a more severe phenotype and higher penetrance [57].

Overall, characterising a pathogenic variant alone does not explain a correlation between genotype and phenotype, and the clinical penetrance of *HMBS* variants is most likely modulated by other genetic and environmental factors [57]. Nevertheless, our report, together with previous variant characterisation at the protein level, demonstrates how the effect of missense substitutions can be grouped into at least two groups where (a) the enzyme has residual activity yielding some turnover of protein, and (b) the enzyme is blocked for turnover in a single intermediate state. These findings can be important in future work exploring alternative treatment options for patients suffering from AIP.

## Conflict of interest

The authors declare no conflict of interest.

## Author contributions

AKA, JPK and HJB conceived the study; JPK and HJB designed experiments and supervised the study; MSC and ML performed experiments; MSC, ML, JPK and HJB analysed data; MSC wrote the first manuscript draft; AKA, JPK and HJB made revisions and finalised the manuscript.

## Supporting information


**Fig. S1.** High‐resolution mass spectra of HMBS‐variant p.R26H. (A) p.R26H measured at denaturing conditions with 1 μM protein. Numbers denote different protein ion‐charge states as [M+nH]^n+^. The high charges and wide charge state distribution indicate that the protein is fully unfolded. Uroporphyrinogen is also detected in denaturing conditions. (B) p.R26H in native‐like conditions at 5 μM concentration. Low charges and narrow charge state distribution show that the protein is folded, and the mutation does not cause an unfolding of the enzyme.
**Fig. S2.** Mass spectra of wt‐HMBS and p.R26H with PBG. (A) Purified wt‐HMBS enzyme showing a mixture of the intermediates E_apo_, E_holo_, ES, ES_2_ and ES_3_. (B) wt‐HMBS with 10× PBG, showing the rapid formation (~1 min) of the ES_4_ intermediate. (C) Incubation of the wt‐HMBS‐PBG mixture for 60 min shows the re‐appearance of the previous intermediates. (D) p.R26H incubated with 10× PBG for 24 h shows ES_2_ form, indicating a lack of activity. Additional peaks correspond to non‐covalent binding of acetate ions. All samples were measured in native‐like conditions in 20 mM NH_4_OAc (pH 6.8) at 5 μM enzyme and 50 μM PBG.
**Fig. S3.** The catalytic activity of wt‐HMBS and p.R26H as a function of substrate (PBG) concentration. Wt‐HMBS and p.R26H measured at standard conditions with 2 μg protein and varying PBG concentrations (0–2000 μM) for a reaction time of 4 min at 37 °C. The specific activity of HMBS was defined as nmol of uroporphyrinogen I/h per mg of enzyme, under the given assay conditions. The data were fitted to Michaelis–Menten kinetics.
**Table S1.** Prediction of secondary structure content. The secondary structure content based on far‐UV CD spectrum recorded at 190–250 nm was estimated in percentage using the DichroWeb software. Multiple unpaired t‐test showed no significant difference between wt‐HMBS and p.R26H.

## Data Availability

The data that support the findings of this study are available from the corresponding author helene.bustad.johannessen@helse-bergen.no upon reasonable request.
