# Supplementary material for: Characterisation of a common hotspot variant in acute intermittent porphyria sheds light on the mechanism of hydroxymethylbilane synthase function
Source: FEBS Open Bio. 2022 Sep 26;12(12):2136–46. doi: 10.1002/2211-5463.13490 (PMC9714363; doi:10.1002/2211-5463.13490)
Supplement: Supplementary file 1 — Fig. S1. High‐resolution mass spectra of HMBS‐variant p.R26H. (A) p.R26H measured at denaturing conditions with 1 μM protein. Numbers denote different protein ion‐charge states as [M+nH]n+. The high charges and wide charge state distribution indicate that the protein is fully unfolded. Uroporphyrinogen is also detected in denaturing conditions. (B) p.R26H in native‐like conditions at 5 μM concentration. Low charges and narrow charge state distribution show that the protein is folded, and the mutation does not cause an unfolding of the enzyme. Fig. S2. Mass spectra of wt‐HMBS and p.R26H with PBG. (A) Purified wt‐HMBS enzyme showing a mixture of the intermediates Eapo, Eholo, ES, ES2 and ES3. (B) wt‐HMBS with 10× PBG, showing the rapid formation (~1 min) of the ES4 intermediate. (C) Incubation of the wt‐HMBS‐PBG mixture for 60 min shows the re‐appearance of the previous intermediates. (D) p.R26H incubated with 10× PBG for 24 h shows ES2 form, indicating a lack of activity. Additional peaks correspond to non‐covalent binding of acetate ions. All samples were measured in native‐like conditions in 20 mM NH4OAc (pH 6.8) at 5 μM enzyme and 50 μM PBG. Fig. S3. The catalytic activity of wt‐HMBS and p.R26H as a function of substrate (PBG) concentration. Wt‐HMBS and p.R26H measured at standard conditions with 2 μg protein and varying PBG concentrations (0–2000 μM) for a reaction time of 4 min at 37 °C. The specific activity of HMBS was defined as nmol of uroporphyrinogen I/h per mg of enzyme, under the given assay conditions. The data were fitted to Michaelis–Menten kinetics. Table S1. Prediction of secondary structure content. The secondary structure content based on far‐UV CD spectrum recorded at 190–250 nm was estimated in percentage using the DichroWeb software. Multiple unpaired t‐test showed no significant difference between wt‐HMBS and p.R26H. [file FEB4-12-2136-s001.pdf]

Supporting information

Table S1. Prediction of secondary structure content. The secondary structure content based on far-UV CD spectrum recorded at 190–250 nm was estimated in percentage using the DichroWeb software. Multiple unpaired t-test showed no significant difference between wt-HMBS and p.R26H.

	Wt-HMBS (%)	p.R26H (%)
**α-helices**	23 ± 1	23 ± 1
**β-sheets**	27 ± 1	28 ± 6
**Turn**	21 ± 0	21 ± 3
**Others**	28 ± 0	28 ± 3

Figures

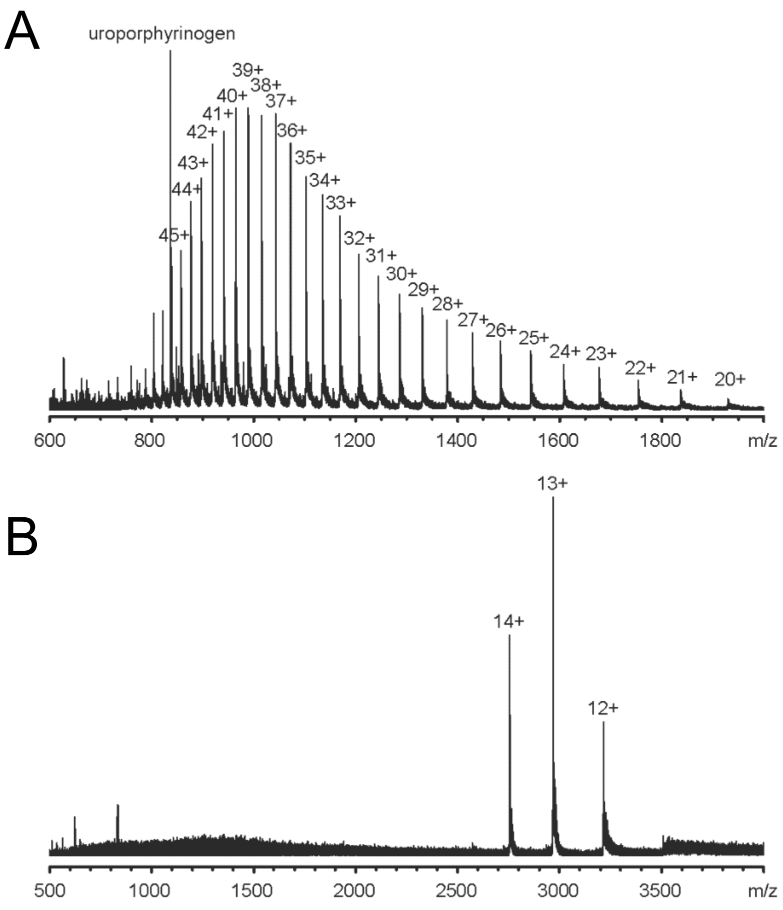

Figure S1. High-resolution mass spectra of HMBS-variant p.R26H. (A) p.R26H measured at denaturing conditions with at 1 μM protein. Numbers denote different protein ion-charge states as $[M+nH]^{n+}$. The high charges and wide charge state distribution indicates that the protein is fully unfolded. Uroporphyrinogen is also detected in denaturing conditions. (B) p.R26H in native-like conditions at 5 μM concentration. Low charges and narrow charge state distribution shows that the protein is folded, and the mutation does not cause unfolding of the enzyme.

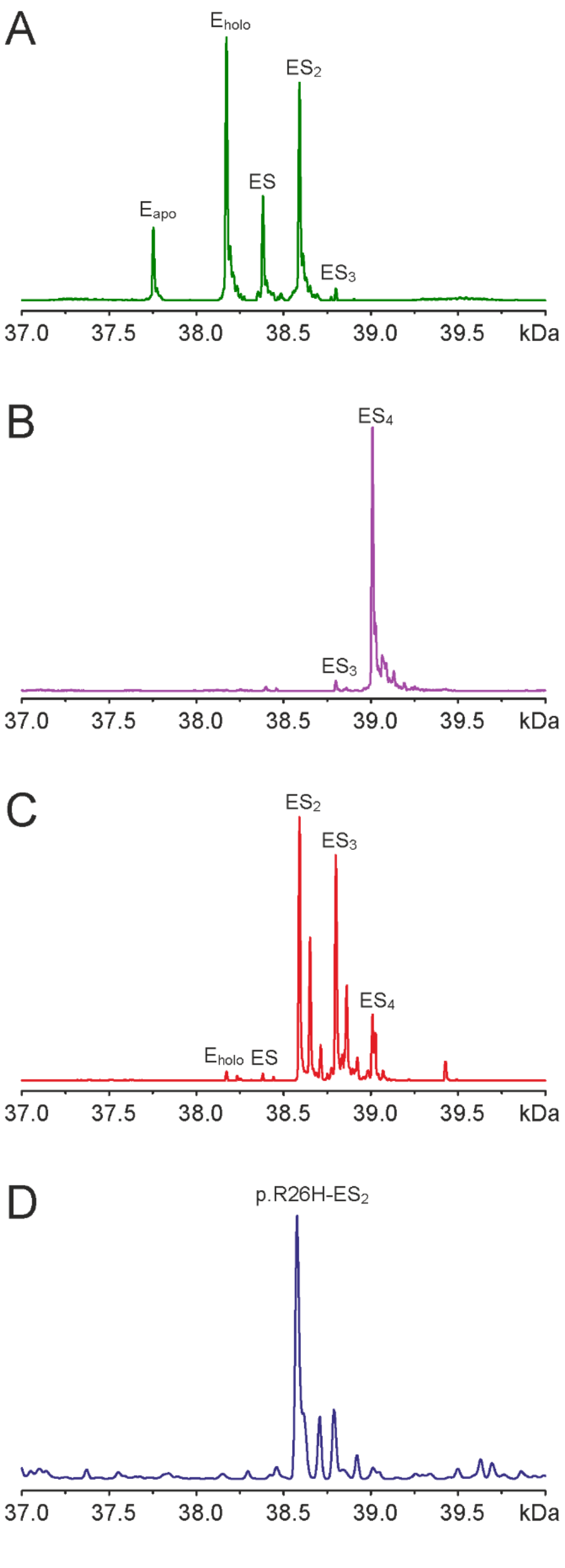

Figure S2. Mass spectra of wt-HMBS and p.R26H with PBG. (A) Purified wt-HMBS enzyme showing a mixture of the intermediates E_{apo}, E_{holo}, ES, ES_2 and ES_3. (B) wt-HMBS with 10× PBG, showing the rapid formation (~1 min) of the ES_4 intermediate. (C) Incubation of the wt-HMBS-PBG mixture for 60 min shows the re-appearance of the previous intermediates. (D) p.R26H incubated with 10× PBG for 24h shows ES_2 form, indicating lack of activity. Additional peaks correspond to non-covalent binding of acetate ions. All samples were measured in native-like conditions in 20 mM NH_4OAc (pH 6.8) at 5 µM enzyme and 50 µM PBG.

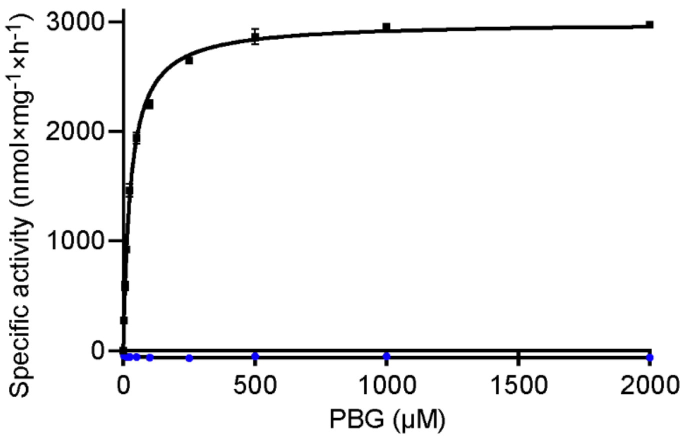

Figure S3: The catalytic activity of wt-HMBS and p.R26H as a function of substrate (PBG) concentration. Wt-HMBS and p.R26H measured at standard conditions with 2 μg protein and varying PBG concentrations (0–2000 μM) for a reaction time of 4 min at 37 °C. The specific activity of HMBS was defined as nmol of uroporphyrinogen I/h per mg of enzyme under the given assay conditions. The data were fitted to Michaelis–Menten kinetics.
